# The Genomic Blueprint of *Salmonella enterica subspecies enterica* serovar Typhi P-stx-12

**DOI:** 10.4056/sigs.3286690

**Published:** 2013-01-20

**Authors:** Su Yean Ong, Chandra Bhan Pratap, Xuehua Wan, Shaobin Hou, Ahmad Yamin Abdul Rahman, Jennifer A. Saito, Gopal Nath, Maqsudul Alam

**Affiliations:** 1 Centre for Chemical Biology, Universiti Sains Malaysia, Penang, Malaysia; 2 Department of Microbiology, Institute of Medical Sciences, Banaras Hindu University, India; 3 Advanced Studies in Genomics, Proteomics and Bioinformatics, University of Hawaii, Honolulu, Hawaii, USA

**Keywords:** *Enterobacteriaceae*, *Salmonella*, Typhi, Gram-negative, host-specific, pathogen, Typhoid Fever

## Abstract

*Salmonella enterica subspecies enterica* serovar Typhi is a rod-shaped, Gram-negative, facultatively anaerobic bacterium. It belongs to the family *Enterobacteriaceae* in the class *Gammaproteobacteria*, and has the capability of residing in the human gallbladder by forming a biofilm and hence causing the person to become a typhoid carrier. Here we present the complete genome of *Salmonella enterica subspecies enterica* serotype Typhi strain P-stx-12, which was isolated from a chronic carrier in Varanasi, India. The complete genome comprises a 4,768,352 bp chromosome with a total of 98 RNA genes, 4,691 protein-coding genes and a 181,431 bp plasmid. Genome analysis revealed that the organism is closely related to *Salmonella enterica* serovar Typhi strain Ty2 and *Salmonella enterica* serovar Typhi strain CT18, although their genome structure is slightly different.

## Introduction

*Salmonella enterica* serovar Typhi is a particular *Salmonella* serovar that causes typhoid fever [1-3]. There are an estimated 20 million cases of typhoid fever and 200,000 deaths from this disease reported each year, worldwide [4,5]. *S. enterica* serovar Typhi belongs to the family *Enterobacteriaceae*. All *Enterobacteriaceae* ferment glucose, reduce nitrates, and are oxidatively negative [6]. In general, *S. enterica* serovar Typhi is motile, produces minimal H_2_S, and is resistant to bile acids [7]. *S. enterica* serovar Typhi has three types of antigens [3], namely the H antigen for motility, specific O antigen for synthesizing lipopolysaccharides and biofilm formation, and Vi antigen which is a capsular polysaccharide that acts as a major virulence factor. This Vi antigen is only specific for *S. enterica* serovar Typhi and is found in Salmonella Pathogenicity Island-7 [8]. In 2003, comparative genomics of *S. enterica* serovar Typhi strains Ty2 and CT18 was carried out by Deng *et al*. [9]. In that study, a half-genome interreplichore inversion in Ty2 relative to CT18 was discovered. It was reported that *S. enterica* serovar Typhi Ty2 does not harbor any plasmid and hence it is susceptible to antibiotics. On the other hand, *S. enterica* serovar Typhi CT18 carries two plasmids with one conferring multidrug resistance. We published the complete genome sequence of *S. enterica* serovar Typhi P-stx-12 earlier last year [10]. This sequencing project helps us to better understand the genome organization and the contribution of the virulence machinery in this pathogen. Here we present a summary of *S. enterica* serovar Typhi P-stx-12 and its unique features, together with the description of the complete genomic sequencing and annotation.

## Classification and features

*S. enterica* serovar Typhi P-stx-12 was isolated from a typhoid carrier in Northern India, Uttar Pradesh, Varanasi in 2009. This serotype is known to inhabit the Peyer’s patches (lymph node) of the small intestine, liver, spleen, bone marrow, bile, and blood stream of infected humans.

Cells of *S. enterica* serovar Typhi P-stx-12 were Gram-negative, motile, rod-shaped, and non-spore forming. This strain grew at an optimum temperature of 35°C-37°C, but could tolerate temperatures between 7°C and 45°C. Strain P-stx-12 is a facultative anaerobe and utilizes glucose as the main carbon source. The pure isolate did not produce cytochrome oxidase but was able to reduce nitrate and break down glucose by pathways for oxidation and fermentation. This strain did not produce urease. In Triple Sugar Iron medium, there was an alkaline/acid reaction with a very small amount of H_2_S production. Indole was not produced in peptone water. The strain was able to ferment glucose and mannitol without production of gas; however lactose and sucrose were not fermented. The strain could be agglutinated by poly O, poly H, factors O9, H-d, and Vi antisera (data not shown).

**Figure 1:**
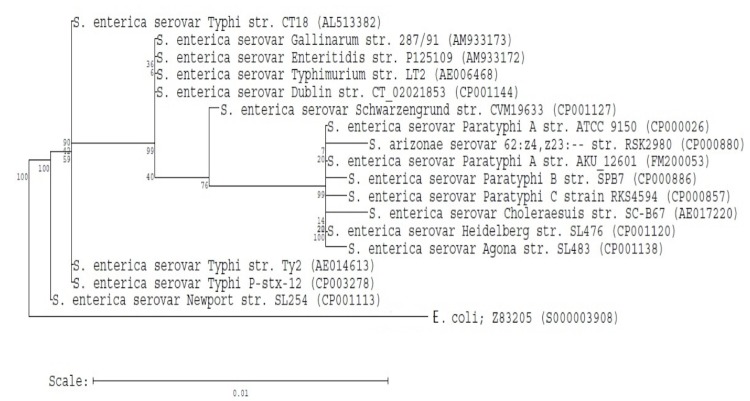
Phylogenetic tree highlighting the position of *Salmonella enterica* serovar Typhi strain P-stx-12 relative to other strains within the *Enterobacteriaceae*. Strains shown are those within the *Enterobacteriaceae* having corresponding GenBank accession numbers. The phylogenetic tree was constructed using Ribosomal Database Project [11] tree builder that utilizes the Weighbor weighted neighbor-joining tree building algorithm [12]. The bootstrap value was 100. *Escherichia coli* strain Z83205 was used as an outgroup.

Figure 1 shows the phylogenetic neighborhood of *S. enterica* serovar Typhi P-stx-12 in a 16S rRNA based tree. There were seven 16S rRNA gene copies in the genome of *S. enterica* serovar Typhi P-stx-12. Two out of the seven copies differed from the rest by having a single base substitution (G to A). Thus, the common gene copy was used for tree building. In relation to others in the genus *Salmonella*, strain P-stx-12 is closely related to *S. enterica* serovar Typhi strain Ty2 and *S. enterica* serovar Typhi strain CT18. The classification and features of this organism are summarized in Table 1.

**Table 1:** Classification and general features of *S. enterica* serovar Typhi P-stx-12

**MIGS ID**	**Property**	**Term**	**Evidence code**^a^
	Current classification	Domain *Bacteria*	TAS [13]
		Phylum *Proteobacteria*	TAS [14]
		Class *Gammaproteobacteria*	TAS [15,16]
		Order *Enterobacteriales*	TAS [17]
		Family *Enterobacteriaceae*	TAS [18-20]
		Genus *Salmonella*	TAS [18,21-23]
		Species *Salmonella enterica*	TAS [23,24]
		Subspecies *Salmonella enterica enterica*	TAS [23,24]
	Gram stain	negative	TAS [6]
	Cell shape	Rod-shape	TAS [6]
	Motility	Motile	TAS [6]
	Sporulation	Non-spore forming	TAS [6]
	Temperature range	7^o^C-45^o^C	TAS [6]
	Optimum temperature	35^o^C-37^o^C	TAS [6]
	Carbon source	Carbohydrates (glucose)	TAS [6]
	Energy source	Chemoorganotrophic	TAS [6]
	Terminal electron receptor	Not reported	
MIGS-6	Habitat	Multi-organ pathogen that inhabits the Peyer’s patches (Lymph node) of the small intestine, liver, spleen, bone marrow, bile and blood stream of infected human.	NAS
MIGS-6.3	Salinity	Survives for days at 0.85% NaCl.	NAS
MIGS-22	Oxygen	Facultative anaerobe	TAS [6]
MIGS-15	Biotic relationship	Human restricted	NAS
MIGS-14	Pathogenicity	Pathogenic	TAS [25,26]
MIGS-4	Geographic location	Uttar Pradesh, Varanasi, India	IDA
MIGS-5	Sample collection time	November 2009	IDA
MIGS-4.1	Latitude	25° 19' 60 N	IDA
MIGS-4.2	Longitude	83° 0' 0 E	IDA
MIGS-4.3	Depth	Not reported	
MIGS-4.4	Altitude	76 (meters)	IDA

a) Evidence codes - IDA: Inferred from Direct Assay; TAS: Traceable Author Statement (i.e., a direct report exists in the literature); NAS: Non-traceable Author Statement (i.e., not directly observed for the living, isolated sample, but based on a generally accepted property for the species, or anecdotal evidence). These evidence codes are from the Gene Ontology project [27]. If the evidence code is IDA, then the property was directly observed for a living isolate by one of the authors or an expert mentioned in the acknowledgements.

## Genome sequencing and annotation

### Genome project history

*S. enterica* serovar Typhi P-stx-12 was selected for sequencing because it was isolated from a typhoid carrier in India, where there is a high rate of typhoid fever cases. This isolate was obtained from a 32-year old male who had been showing persistent high titers for Widal test and Vi antibody for more than one year. DNA isolation was carried out at Banaras Hindu University. This genome sequence was first published in April 2013 [10]. A summary of the project information is shown in Table 2.

**Table 2:** Genome sequencing project information

**MIGS ID**	**Property**	**Term**
MIGS-31	Finishing quality	Finished
MIGS-28	Libraries used	One 454 paired-end library (4-kb insert size), one Illumina library
MIGS-29	Sequencing platforms	Illumina GA IIx, 454 GS FLX Titanium
MIGS-31.2	Fold coverage	100× Illumina, 18× pyrosequencing
MIGS-30	Assemblers	Newbler, Burrows-Wheeler Alignment
MIGS-32	Gene calling method	Glimmer, RNAmmer, tRNAscan-SE
	Genbank ID	CP003278 (Chromosome) CP003279 (Plasmid)
	Genbank Date of Release	February 1, 2012
	Project relevance	Biotechnology, Pathway, Pathogenic

### Growth conditions and DNA isolation

The stool specimen of strain P-stx-12 was collected from a known chronic typhoid carrier patient. For the isolation of the bacterium, 5 gm of freshly passed unpreserved stool was sieved through a gauze piece to remove the coarse particles. The filtrate was centrifuged at 4,000 rpm for 5 min. The pellet was washed twice with Phosphate Buffered Saline, pH 7.2 and suspended in selenite F broth (50 ml) for enrichment with some modified technique (under process of patenting). After overnight incubation, the broth was examined for turbidity and subcultured on deoxycholate citrate agar and MacConkey agar.

Extraction of genomic DNA was carried out using a Phenol-Chloroform and Proteinase K method with some modification [28]. The DNA preparation was checked by PCR amplification of the flagellin (*fliC*) gene of *S. enterica* serovar Typhi [29,30] and 16S rRNA gene [31].

### Genome sequencing and assembly

Whole-genome sequencing was performed with a combined strategy of 454 and Illumina sequencing technologies. A 4-kb paired-end library was constructed according to the manufacturer’s instructions (454). A total of 242,499 reads were generated using the GS FLX Titanium system, giving ~18× coverage of the genome. Initial assembly of 97.09% of the reads using the Newbler assembler (Roche) resulted in ~200 large contigs within 11 scaffolds. A total of ~500 Mb of 3-kb mate-pair sequencing data were generated to reach a depth of 100× coverage with an Illumina GA IIx. These sequences were mapped to the scaffolds using the Burrows-Wheeler Alignment (BWA) tool [32]. A majority of the gaps within the scaffolds were filled by local assembly of 454 and Illumina reads. The remaining gaps were filled by sequencing the PCR products of the gaps using an ABI 3730xl capillary sequencer. The putative sequencing errors were verified by the coverage of 454 and Illumina reads.

### Genome annotation

Annotation of the *S. enterica* serovar Typhi P-stx-12 genome was done using a combination of ISGA (Integrative Services for Genomic Analysis) [33] and the DIYA (Do-It-Yourself Annotator) pipeline [34], which comprises of Glimmer [35], tRNAscan-SE [36], RNAmmer [37], BLAST [38], and Asgard [39]. RPS-BLAST searches against the Clusters of Orthologous Groups (COG) database enabled assignment of COG functional categories to the ORFs. CLC Genomics Workbench was used to further improve and check the annotation results. Frameshifts and partial gene fragments that indicate potential pseudogenes were identified by the NCBI Submission Check tool and manually verified. Protein coding genes were searched against the NCBI RefSeq database using BLASTP [40]. Clustered Regularly Interspersed Short Palindromic Repeats (CRISPR) regions were identified using the CRISPR Finder program [41]. PHAST (PHAge Search Tool) [42] was used to search for prophage sequences within the genome. Potential genomic islands were identified using the IslandViewer web server [43]. Comparison between different *S. enterica* serovar Typhi strains was done using progressiveMauve [44].

## Genome properties

**Figure 2:**
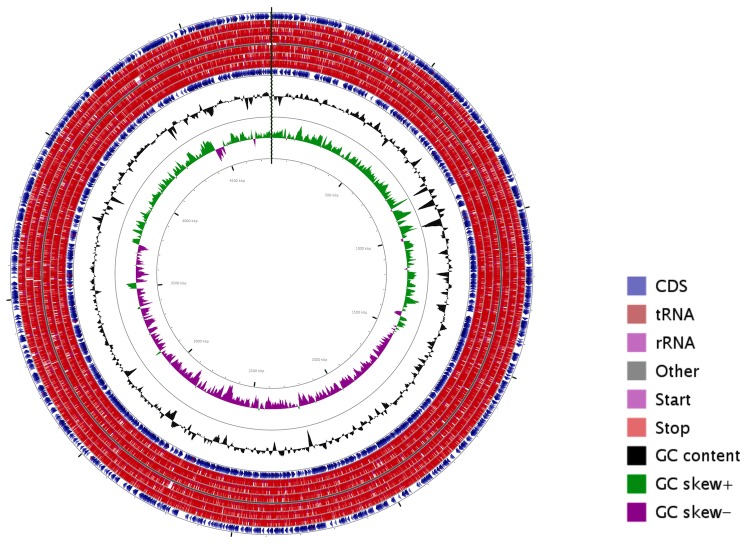
Circular map of the *Salmonella enterica* serovar Typhi P-stx-12 chromosome. From the inside to outside, the first and second circles show GC skew and G+C content respectively. The third circle shows the CDS, tRNA and rRNA in the reverse strand; the fourth circle shows the CDS, tRNA, rRNA in the forward strand. This figure was generated by CGView [45].

**Figure 3:**
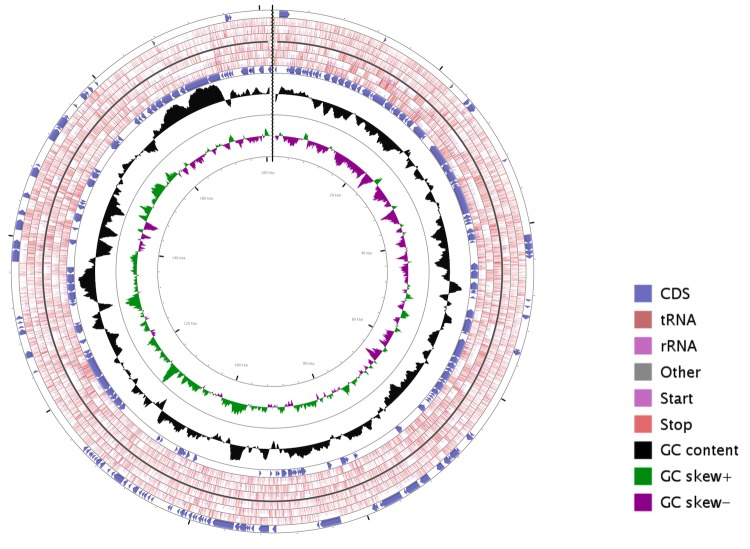
Circular map of the *Salmonella enterica* serovar Typhi P-stx-12 plasmid. From the inside to outside, the first and second circles show GC skew and G+C content respectively. The third circle shows the CDS, tRNA and rRNA in the reverse strand; the fourth circle shows the CDS, tRNA, rRNA in the forward strand. This figure was generated by CGView [45].

The complete genome of *S. enterica* serovar Typhi P-stx-12 contains a single circular chromosome of 4,768,352 bp with a GC content of 52.1%, and a circular plasmid of 181,431 bp with a GC content of 46.4% (Figure 2 and Figure 3). The chromosome consists of 4,885 predicted genes, of which there are 4,691 protein-coding genes, 22 rRNA genes, and 76 tRNA genes. Specific COGs were assigned to 75.34% of the genes in the chromosome, and 25% of these genes were also assigned with enzyme classification numbers which were involved in 268 metabolic pathways. The properties and statistics of the genome are summarized in Tables 3 and 4. The plasmid harbors 234 protein-coding genes, with 187 annotated as hypothetical proteins with unknown function. The remaining genes were grouped into specific COGs, the majority of which fell into the category of information storage and processing with respect to replication, recombination and repair.

**Table 3:** Genome statistics

**Attribute**	**Value**	**% of total^a^**
Genome size (bp)	4,768,352	100.00
DNA coding region (bp)	4,018,014	84.26
DNA G+C content (bp)	2,484,311	52.10
Total genes^b^	4,885	100.00
RNA genes	98	2.00
Protein-coding genes	4,691	96.03
Pseudogenes	96	1.97
Genes in paralog clusters	623	13.28
Genes assigned to COGs	3,534	75.34
Genes with signal peptides	388	8.27
Genes with transmembrane helices	1,096	23.36
CRISPR repeat	1	

a) The total is based on either the size of the genome in base pairs or the total number of protein coding genes in the annotated genome.

b) Also includes 96 pseudogenes

**Table 4:** Number of genes associated with the 25 general COG functional categories

**Code**	**Value**	**%age^a^**	**Description**
J	182	3.9	Translation
A	1	0.0	RNA processing and modification
K	307	6.5	Transcription
L	191	4.1	Replication, recombination and repair
B	0	0.0	Chromatin structure and dynamics
D	33	0.7	Cell cycle control, mitosis and meiosis
Y	0	0.0	Nuclear structure
V	48	1.0	Defense mechanisms
T	181	3.9	Signal transduction mechanisms
M	246	5.2	Cell wall/membrane biogenesis
N	122	2.6	Cell motility
Z	0	0.0	Cytoskeleton
W	1	0.0	Extracellular structures
U	139	3.0	Intracellular trafficking and secretion
O	156	3.3	Posttranslational modification, protein turnover, chaperones
C	262	5.6	Energy production and conversion
G	352	7.5	Carbohydrate transport and metabolism
E	349	7.4	Amino acid transport and metabolism
F	89	1.9	Nucleotide transport and metabolism
H	176	3.8	Coenzyme transport and metabolism
I	85	1.8	Lipid transport and metabolism
P	193	4.1	Inorganic ion transport and metabolism
Q	65	1.4	Secondary metabolites biosynthesis, transport and catabolism
R	421	9.0	General function prediction only
S	355	0.1	Function unknown
-	1157	24.7	Not in COGs

a) The total is based on the total number of protein coding genes in the annotated genome.

### Paralog clusters

**Figure 4:**
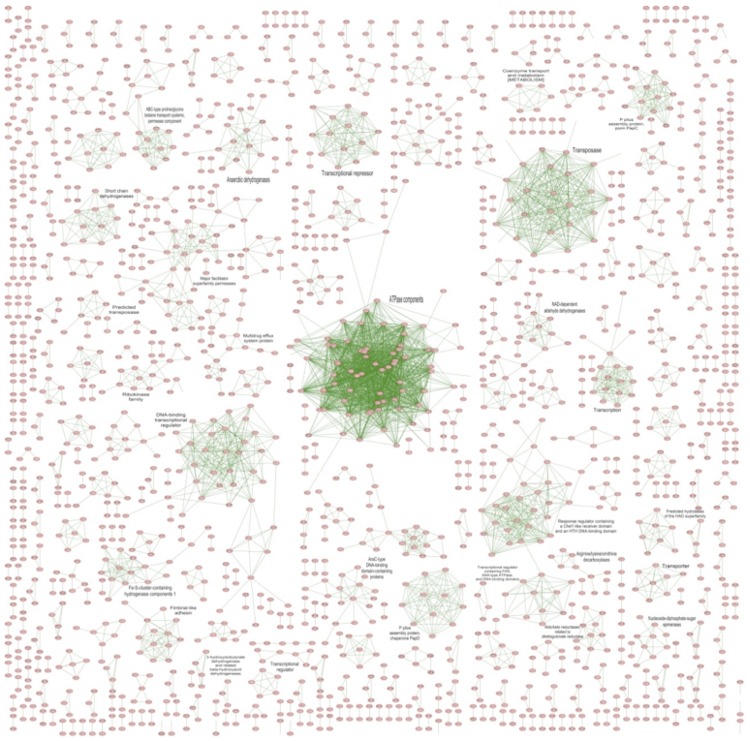
Paralog network of functional families in the *S. enterica* serovar Typhi P-stx-12 genome.

In order to identify paralog families, BLASTP was used to calculate all possible protein homologs in the *S. enterica* serovar Typhi P-stx-12 genome. Homologs that had at least 30% shared amino acid similarity were selected. Paralog pairs were imported into the *S. enterica* serovar Typhi P-stx-12 database in Pathway Studio as a new type of interaction called “Paralog” [46]. Protein functional families were identified as clusters in the global Paralog network using the direct force layout algorithm. The biological function was assigned to each paralog cluster based on the functional annotation of the protein (Figure 4). The major paralog clusters identified include ATPase components that are mainly involved in transport systems, transcriptional regulator, transcriptional repressor, transposases, major facilitator superfamily permeases, response-regulator containing CheY-like receiver domain and an HTH DNA binding domain, P-pilus assembly proteins, multidrug efflux system proteins, and fimbrial-like adhesins.

## Insights into the genome

### Comparisons with other fully sequenced *S. enterica* serovar Typhi genomes

**Figure 5:**
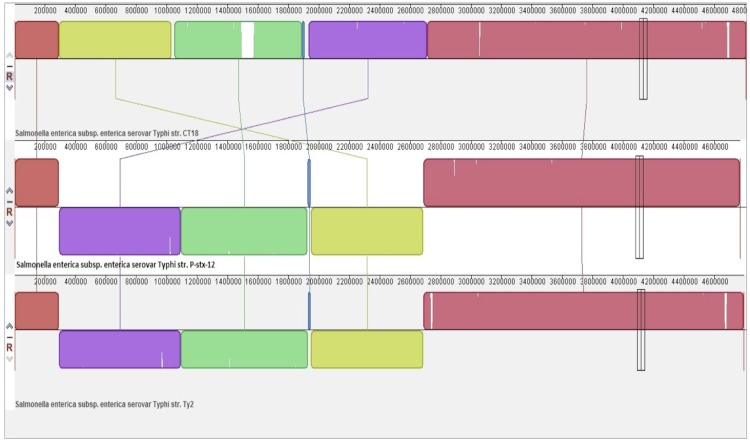
Alignment of the *S. enterica* serovar Typhi CT18, *S. enterica* serovar Typhi P-stx-12, and *S. enterica* serovar Typhi Ty2 genomes using progressive Mauve [44]. Colored blocks in the first genome are connected by lines to similar colored blocks in the second and third genomes. Inverted regions in *S. enterica* serovar Typhi P-stx-12 and *S. enterica* serovar Typhi Ty2 are presented as blocks below the center line of the genome. Lines indicate regions in each genome that are homologous.

The genome of *S. enterica* serovar Typhi P-stx-12 was compared with the other two published *S. enterica* serovar Typhi genomes, CT18 (isolated from Vietnam) and Ty2 (isolated from Russia). Comparison between these three genomes revealed that the coding genes of *S. enterica* serovar Typhi P-stx-12 were 84% similar to those of CT18 [47] and Ty2 [9]. The genome organization of these three strains is shown in Figure 5. The location of the genes in strains P-stx-12 and Ty2 are identical. Both have three blocks of genes that are inverted from strain CT18. Our observations are in agreement with the work of Deng *et al*. [9], where they discovered that half of the Ty2 genome was inverted relative to the CT18 genome. Nevertheless, most of the genes have the same function, indicating that these are the possible housekeeping genes which maintain the survival of this pathogen. Besides that, this P-stx-12 strain has one plasmid which shares 169 orthologous CDSs with pHCM1, the plasmid belonging to CT18 (Genbank accession number AL513383). pHCM1 is a conjugative plasmid which encodes resistance to antimicrobial agents and heavy metals; similar to IncHI plasmid R27. This further supports the hypothesis that the presence of a plasmid signifies a dynamic link between resistance and pathogenicity. Indeed, it was reported that the stable maintenance of IncHI1 plasmids in *S. enterica* serovar Typhi occurred throughout the development of antibiotic resistance in *S. enterica* serovar Typhi [48]. It is worth noting that the plasmid of P-stx-12 carries genes encoding the tetracycline resistance protein and tetracycline repressor protein TetR, possibly conferring drug resistance to this strain. This resistance protein is also found in strain CT18. On the other hand, the number of pseudogenes in this genome appears to be only 96, which is less than those in *S. enterica* serovar Typhi CT18 and *S. enterica* serovar Typhi Ty2 (> 200).

### Genomic Islands (GIs) and Salmonella Pathogenicity Island (SPIs)

**Figure 6:**
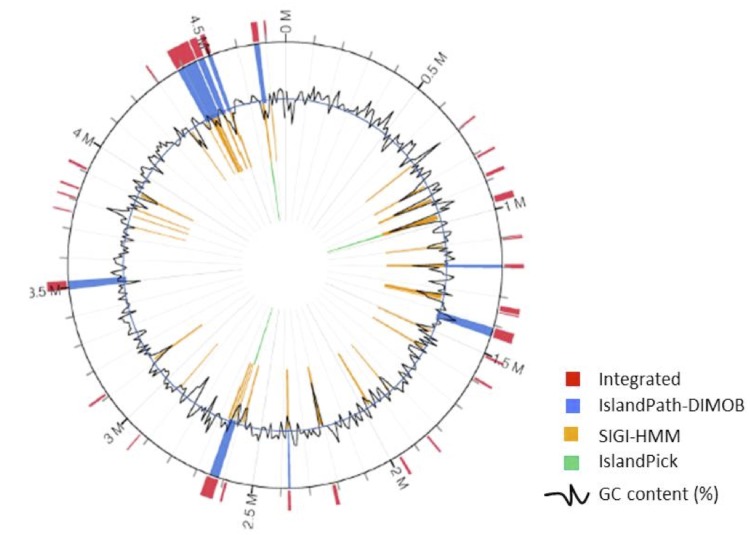
Genomic islands as predicted using IslandViewer. Predicted genomic islands are colored within the circular image based on the tool IslandPath-DIMOB, SIGI-HMM, IslandPick, and an integration of the three tools.

There are 31 possible genomic islands (GIs) as predicted by IslandViewer (Figure 6). Analysis of these GIs revealed that most of the genes within the islands encode for hypothetical proteins. Eight Salmonella Pathogenicity Islands (SPI-11, SPI-2, SPI-16, SPI-6, SPI-8, SPI-4, SPI-7 and SPI-10) were found to be embedded in these GIs, whereas the rest of the SPIs spanned between the GIs. Interestingly, the proteins found in SPI-8 are located next to the proteins of SPI-13, which is not classified as one of the predicted GIs. Three GIs within the coordinate 4,376,723 to 4,508,803 make up the total region for SPI-7.

**Figure 7:**
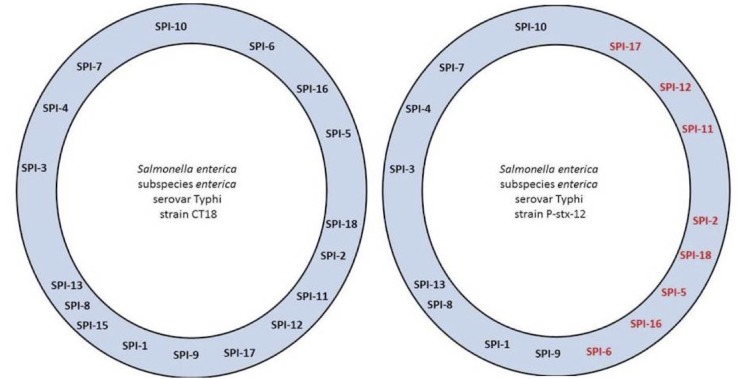
Distribution of SPIs in *S. enterica* serovar Typhi CT18 and *S. enterica* serovar Typhi P-stx-12.

A comparison between the SPIs found in strains CT18 and P-stx-12 revealed that the location of several SPIs in both genomes is different (Figure 7). Indeed, the orientation for SPI-6, SPI-16, SPI-5, SPI-18, SPI-2, SPI-11, SPI-12, and SPI-17 was inverted in both genomes. These SPIs fall within the inverted genomic regions shown in Figure 5.

### Prophage Regions

Prophage are one of the diverse mobile genetic elements that are acquired through horizontal gene transfer. These prophage genes are involved in lysogenic conversion. PHAST (PHAge Search Tool) was used to identify the prophage regions of *S. enterica* serovar Typhi P-stx-12. Based on the analysis, five predicted prophage regions (three intact, two partial) were identified in the genome. The three intact prophage regions have the size of 44.2 kb, 50.8kb, and 68.2 kb, respectively. These regions consist of a total of 165 coding sequences for the phages phage_Gifsy_2 and Enterobacteria_phage_Fels2. In comparison, *S. enterica* serovar Typhi CT18 and *S. enterica* serovar Typhi Ty2 each have eight predicted prophage regions. Out of the eight regions, only four intact regions (247 proteins) were found in *S. enterica* serovar Typhi CT18 whereas three intact regions (170 proteins) were found in *S. enterica* serovar Typhi Ty2. The phage regions of *S. enterica* serovar Typhi P-stx-12 are the same types as those found in *S. enterica* serovar Typhi Ty2, while *S. enterica* serovar Typhi CT18 carries an additional phage region of the Enterobacteria_phage_SfV type. A summary of the prophage regions in each genome is shown in Table 5.

**Table 5:** Prophage regions identified in *S. enterica* serovar Typhi P-stx-12, *S. enterica* serovar Typhi CT18, and *S. enterica* serovar Typhi Ty2

**Organism**	**Region length**	**# CDS**	**Region Position**	**Phage**	**% GC**
***S. enterica* serovar Typhi P-stx-12**	44.2 kb	59	1924908-1969179	Phage_Gifsy_2	50.35
	50.8 kb	49	3478537-3529349	Enterobacteria_phage_Fels2	52.21
	68.2 kb	57	4424417-4492645	Enterobacteria_phage_Fels2	50.07
***S. enterica* serovar Typhi CT18**	44.3 kb	64	1008698-1053060	Phage_Gifsy_2	50.19
	**59.7 kb**	**74**	**1879760-1939495**	**Enterobacteria_phage_SfV**	**48.12**
	50.8 kb	49	3504242-3555052	Enterobacteria_phage_Fels2	52.21
	48.1 kb	60	4459144-4507270	Enterobacteria_phage_Fels2	51.15
***S. enterica* serovar Typhi Ty2**	44.2 kb	63	1928058-1972330	Phage_Gifsy_2	50.35
	50.8 kb	48	3489900-3540712	Enterobacteria_phage_Fels2	52.20
	45.1 kb	59	4446021-4491188	Enterobacteria_phage_Fels2	51.36

### CRISPR Region

By using the CRISPR Finder tool, one CRISPR repeat region with a length of 394 bp was identified in the *S. enterica* serovar Typhi P-stx-12 genome. The CRISPR region starts at the position 2,900,675 and ends at the position 2,901,069 with 6 spacers in between. The confirmed CRISPR has the following direct repeat consensus sequence: CGGTTTATCCCCGCTGGCGCGGGGAACAC. Strains CT18 and Ty2 also have a single CRISPR repeat region with the lengths of 385 bp and 394 bp, respectively. The location for the CRISPR region of all three strains falls within the region of 2.9 Mbp on the chromosome. All the strains have 6 spacers and share the common direct repeat consensus sequence. It is worth noting that the CRISPR region, including the length and the spacer sequence, of *S. enterica* serovar Typhi P-stx-12 is exactly identical to *S. enterica* serovar Typhi Ty2. It suggests a strong evidence of their evolutionary relevance and shows that the CRISPR region in *S. enterica* serovar Typhi is conserved. As CRISPRs function as a prokaryotic immune system and confer resistance towards plasmids and phages (thus interfering with the spread of antibiotic resistance and virulence factors), it is reasonable to find only one CRISPR with very few spacers in this pathogen as compared to other bacterial strains that are not pathogenic [49].
